# Biodistribution of Cy5-labeled Thiolated and Methylated Chitosan-Carboxymethyl Dextran Nanoparticles in an Animal Model of Retinoblastoma

**DOI:** 10.18502/jovr.v17i1.10171

**Published:** 2022-01-21

**Authors:** Elham Delrish, Fariba Ghassemi, Mahmoud Jabbarvand, Alireza Lashay, Fatemeh Atyabi, Masoud Soleimani, Rassoul Dinarvand

**Affiliations:** ^1^Translational Ophthalmology Research Centre, Farabi Eye Hospital, Tehran University of Medical Sciences, Tehran, Iran; ^2^Retina & Vitreous Service, Farabi Eye Hospital, Tehran University of Medical Sciences, Tehran, Iran; ^3^Nanotechnology Research Centre, Faculty of Pharmacy, Tehran University of Medical Sciences, Tehran, Iran; ^4^Department of Pharmaceutics, Faculty of Pharmacy, Tehran University of Medical Sciences, Tehran, Iran; ^5^Department of Hematology, School of Medical Sciences, Tarbiat Modares University, Tehran, Iran

**Keywords:** Biodistribution, Carboxymethyl Dextran, Chitosan, Cy5-Labeled, Nanoparticles, Retinoblastoma

## Abstract

**Purpose:**

The use of more potent medicine for local chemotherapy of retinoblastoma in order to minimize local and systemic adverse effects is essential. The main goal of this investigation was to assess the biodistribution of thiolated and methylated chitosan-carboxymethyl dextran nanoparticles (CMD-TCs-NPs and CMD-TMC-NPs) following intravitreal (IVT) injection into rat eyes with retinoblastoma.

**Methods:**

An ionic gelation method was used to fabricate Cy5-labelled CMD-TCs-NPs and CMD-TMC-NPs. The NPs were characterized. Cellular internalization of Cy5-labelled NPs was investigated using confocal microscopy and the absorption of labeled NPs was quantified by flow cytometry in human retinoblastoma (Y79) cells. In addition, the Cy5-labeled distribution of nanoparticles in the posterior segment of the eye was histologically imaged by confocal microscopy after IVT injection of NPs into the eyes of rats with retinoblastoma.

**Results:**

CMD-TCs-NPs and CMD-TMC-NPs showed a mean diameter of 34 ± 3.78 nm and 42 ± 4.23 nm and zeta potential of +11 ± 2.27 mV and +29 ± 4.31mV, respectively. The in vivo study of intraocular biodistribution of Cy5-labeled CMD-TCs-NPs and CMD-TMC-NPs revealed that there is more affinity of CMD-TCs-NPs to the retina and retinoblastoma tumor after IVT administration while methylated chitosan nanoparticles are immobilized in the vitreous and are not able to reach the retina even after 24 hr.

**Conclusion:**

The ionic gelation technique was efficient in synthesizing a biocompatible polymeric nanosystem for drug delivery into the posterior segment of the eye. The current study demonstrated increased ocular bioavailability of CMD-TCs-NPs relative to CMD-TMC-NPs in retinoblastoma induced rat eyes.

##  INTRODUCTION

The blood–retinal barrier prevents large molecules from passing into the retina from the blood and choriocapillaris.^[1]^ The presence of efflux transporters and the pigmented structure of the choroid are the major limiting factors affecting therapeutic molecule penetration from the choroid to the retina and subsequently into the vitreous.^[2,3]^ Sclera mainly limits the delivery of lipophilic drugs. The effect of the molecular radius however is greater than that of lipophilicity, which affects the scleral permeability of the drug.^[1]^ Vitreous, which consists mostly of 99% water, also contains only a few solid components, such as collagen and glycosaminoglycans.^[4]^ The vitreous poses a substantial barrier to injectable therapeutic molecules, especially to the diffusion of suspended solids or combinations of high molecular weight.^[5]^

Intravitreal (IVT) injection is the most popular method for delivering drugs into the posterior portion of the eye. It delivers the needed therapeutic concentration of the drug to the posterior segment with minimal but considerable hazards.^[6,7,8]^ With recent developments in nanocarriers, polymeric carriers are being employed in facilitating drug delivery to the eye to improve the drug's bioavailability.^[9,10,11,12]^ Natural polysaccharides are attractive for the formulation of ocular medications because they are nontoxic, economical, available, generally biodegradable and biocompatible, and usually amenable to chemical modification to fabricate new derivatives.^[13,14,15,16,17,18]^ Chemical modifications have been recently used to fabricate derivatives with improved properties in terms of mucoadhesion, increased ocular bioavailability, and drug solubilization.^[19,20,21,22,23]^

The bioavailability of NPs in the retina can be enhanced using this technique. It is proven that PEG-coated polystyrene NPs with neutral surface charge up to the size of 750 nm could freely diffuse through bovine vitreous to reach the retina. Diffusion coefficients in nanoparticles were found to be greater at 100–500 nm rather than at 750 nm. Carboxylic groups coating was used to fabricate negatively charged beads, which were able to readily diffuse through the vitreous. Negatively charged nanoparticles, on the other hand, are more impacted by size than neutrally charged NPs, as a negative-500nm-particle was unable to efficiently disperse through vitreous fluid.^[26]^ When nanoparticles made of human serum albumin (HSA), hyaluronic acid, or a combination of the two are injected intravitreally, they can reach the retina. Polyethylene imine nanoparticles with positive surface charge cannot spread through the vitreous when intravitreally injected and are therefore not beneficial for IVT route. Nanoparticles fabricated from glycosylated chitosan (200–500 nm) can reach the retina when intravitreally injected but are not able to penetrate inner limiting membrane.^[27]^

To the best of our knowledge, no study has been performed to investigate the bioavailability of Cy5 fluorescent dye oligonucleotide labeled thiolated and methylated chitosan nanoparticles following IVT injection in the eyes of rats with retinoblastoma. Therefore, in this investigation, we characterized the effects of the surface charge of thiolated and methylated chitosan NPs on the diffusion and tissue distribution after a single IVT injection into the retinoblastoma bearing rat eyes.

##  METHODS

### Materials

Medium-molecular-weight chitosan (Cs) with a degree of deacetylation of about 89% was purchased from Primex (Karmoy, Norway). N-ethylcarbodiimide hydrochloride (EDC), N-hydroxysuccinimide (NHS), carboxymethyl dextran (CMD) sodium salt (10–20 KD, 1.1–1.5 mmol carboxyl/g), Ellman's reagent, RPMI-1640 tissue culture medium, fetal bovine serum (FBS), and dialysis tubing (molecular weight cut-off 2, and 12 kDa)`1 were purchased from Sigma-Aldrich (Missouri, USA). N-Methyl-2-pyrrolidone (NMP), sodium chloride, hydrochloric acid, and sodium hydroxide (NaOH) were all purchased from Merck (Darmstadt, Germany). The human retinoblastoma cell line (Y79). All chemicals were of analytical grade.

### Synthesis and Characterization of TMC

TMC was synthesized according to the method reported by Sieval et al.^[28]^ The degree of quaternization (% DQ) was distinguished using ^1^H NMR spectrum of TMC which was prepared by a 600 MHz spectrometer (Bruker-Biospin, Germany). The %DQ was estimated by the following formula:

DQ = [[(CH3)3 / [H] × 1/9] × 100,

where DQ is the level of quaternization; [(CH3)3] is the integral of chemical shift of the hydrogens of N+(CH3)3 groups at 3.4 ppm; and [H] is the integral of H-1 peaks between 4.7 and 5.7 ppm.^[29]^

### Synthesis and Characterization of TMC-Cysteine Conjugates:

The method of synthesis was according to Margit et al.^[30]^ In the first step, 100 milligrams (mgr) of synthesized TMC was dissolved in 5 ml of distilled water (DI) and then 200 mgr of cysteine was added and then mixed until dissolved. In the second stage, EDC and NHS were added. The mixture was then incubated for 3 hr in the dark under continuous stirring at room temperature and the pH was balanced to 5. Afterward, the solution was dialyzed (membrane dialysis MW cut-off = 2 kDa) using 1 mM HCl for three days at 4ºC. Eventually, the solution was lyophilized to obtain a powdery substance (TMC-cys) and stored at 4ºC. The amount of free thiol groups attached on the TMC backbone was determined by photometry with Ellman's reagent. The thioglycolic acid standards curve was used to determine the quantitative amount of thiol groups.^[31]^ FT-IR spectra of TCs were prepared with an FTIR spectrophotometer (Vectore 22, Germany).

### Preparation of CMD-TCs Nanoparticles

The nanoparticles were fabricated by a simple coacervation technique.^[32]^ Carboxymethyl dextran (CMD) was used as the cross-linking agent. Nanoparticles were prepared by adding CMD solutions to TCs or TMC solutions. Then, an instant vortex stirring was executed and samples were incubated at room temperature for 2 hr.

### Nanoparticles Characteristics

The particle size of the nanoparticles was distinguished by applying dynamic light scattering on a Malvern Zetasizer Nano-ZS (Worcestershire, United Kingdom). A Zetasizer Nano series (Malvern Instruments) was performed to determine the surface charge of the NPs. Field emission scanning electron microscopy (FESEM; ZEISS) and transmission electron microscopy (TEM, Zeiss, EM 900) were used to study the morphology of nanoparticles.

### In Vitro Cellular Uptake of Nanoparticles

Qualitative cellular uptake of Cy5-loaded NPs was investigated with a confocal laser scanning microscope (Nikon, Eclipse).^[33]^ For this purpose, the Y79 cells were cultured in 6-wells at the density of 2 × 10^5^ cells per well. When the cells reached confluence, the cells were then incubated with Cy5-labeled TMC-CMD-NPs and TMC-cys-CMD-NPs suspension to track their uptake in Y79 cells. As a result of this procedure, the nanoparticles were well-dispersed in the culture medium at concentrations of 100 μg/ml. Nanoparticle dispersions were incubated at 37ºC in a 5% CO ${}_{2}$ atmosphere for 2 hr. After aspiration of the medium, the cells were rinsed with 10 ml of cold phosphate buffered saline (PBS) (pH7.4) to eliminate any traces of nanoparticles remaining in the medium. Then, the cells were fixed with 2% paraformaldehyde for 10 min at room temperature and stained with DAPI (4',6-Diamidino-2-phenylindole dihydrochloride). The fluorescence of the Cy5-labeled nanoparticles was monitored applying a confocal microscope (excitation 640.8 nm/emission 662–737 nm).

### Quantifying Level of NPs Cellular Uptake By Flow Cytometry

The cellular internalization of Cy5-labeled CMD-TMC-NPs and CMD-TCs-NPs were reconfirmed and compared by flow cytometric analysis in the Y79 cells. To execute, the cells were cultured in a 6-well plate at a density of 250 × 10^4^ cells/well. After 24 hr of incubation, the cells were treated with Cy5-labeled NPs at 37ºC for 2 hr. After the incubation, cells were washed with PBS and analyzed for intracellular fluorescence of Cy5-labeled NPs using BD FACS Calibur flow cytometer (BD Biosciences, San Jose, CA, USA).^[34]^

### Rat Xenograft Model of Retinoblastoma

For this study, 10 Wistar albino rats (male, two months old, purchased from Pasteur Institute, Karaj, Iran) were used. All rats were treated in accordance with the ARVO (Association for Vision and Ophthalmology Research) Declaration on the Procedure of Animals in Ophthalmic and Vision Research, approved by the University of Medical Sciences of Tehran. Surgeries were performed by the same surgeon (FG). The rats were immunosuppressed with daily injections of Cyclosporin A (CsA) (SandimmunⓇ; Novartis). Approximately 1 × 10^6^ Y79 cells were intravitreally injected to the rat eyes.^[35]^ After retinoblastoma tumor formation, Cy5-labeled TMC-CMD-NPs and TMC-cys-CMD-NPs (100 µg/ml) was intravitreally injected. The control eyes received IVT normal saline as same concentration. All the animals were euthanized 24 hr after the IVT injection of Cy5-labeled nanoparticles and enucleation was performed on them. Afterward, tissues were cut into 5-µm thick layers using a microtome for investigation of qualitative ocular uptake and biodistribution of Cy5-labeled NPs, which was done with a confocal laser scanning microscope (Nikon, Eclipse).

##  RESULTS

### Nanoparticles Characteristics

**Figure 1:**
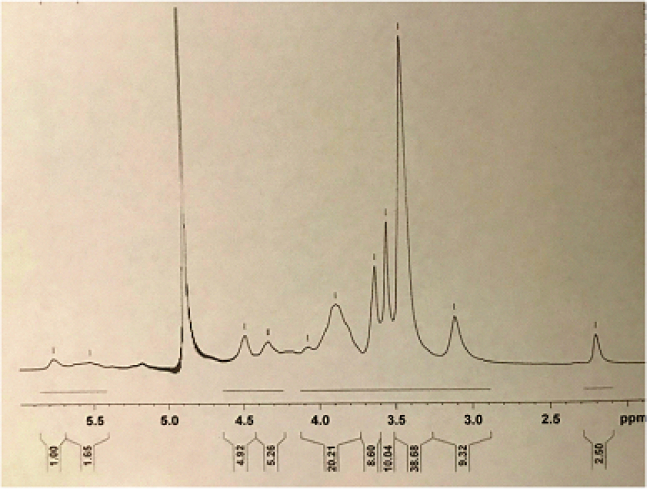
FT-IR spectra of Cs, TMC, and TCs.

**Figure 2:**
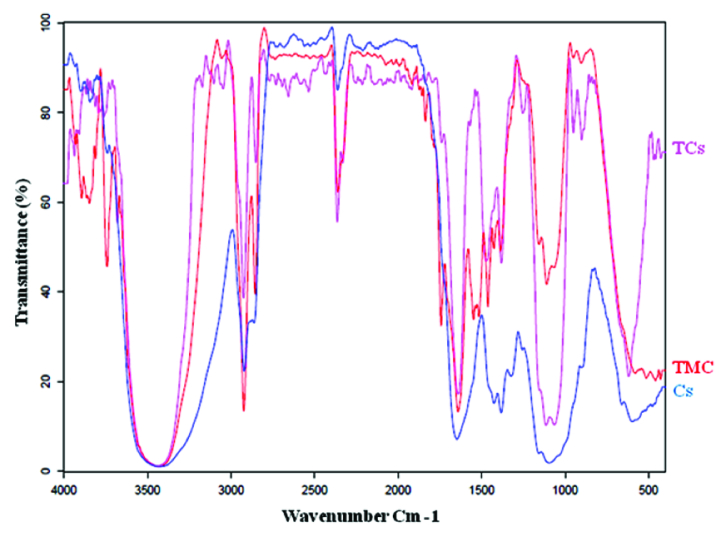
^1^H-NMR spectrum of TMC in D2O.

**Figure 3:**
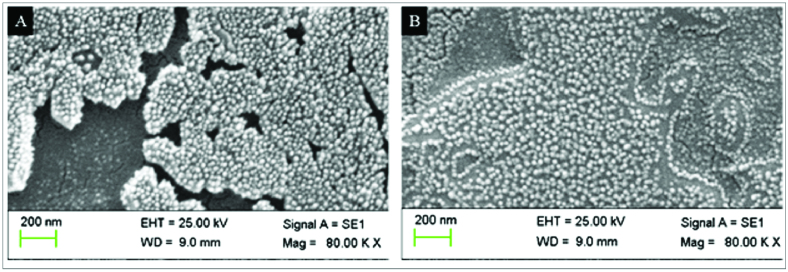
SEM images of CMD-TMC-NPs (A) and CMD-TCs-NPs (B).

The ^1^H NMR spectrum of TMC is shown in Figure 1. In the ^1^H NMR spectrum of TMC, the signals at 3.3 to 3.8 ppm were attributed to the methyl group at the N,N,N-trimethylated site ([H3]–[H6]).^[36]^ FTIR spectroscopy is an efficient tool for the investigation of the physicochemical attributes of polysaccharide. In this study, the syntheses of TMC and TCs were corroborated by the FTIR spectra illustrated in Figure 2. The TMC-cys conjugate was synthesized by the development of amide bonds between the amino group of methylated chitosan and carboxylic acid group of cysteine. Meanwhile, for TMC the peak at 1470 cm ${}^{-}$ ^1^ corresponded to the characteristic absorption of N–CH3. The peak at around 1250 cm ${}^{-}$ ^1^ in the spectra of compound was accredited to the C–SH stretching band. Also, the spectra of thiolated-chitosan displayed two powerful characteristic absorptions at 1641 cm ${}^{-}$ ^1^ and 2500 cm ${}^{-}$ ^1^ which were attributed to the C = O double bonds of the amido group and stretching vibration of –SH, respectively [Figure 2].^[37,38]^ Furthermore, the degree of substitution of thiols using Ellman's protocol was determined as 11%. In addition, CMD-TCs-NPs and CMD-TCs-NPs had diameters of 34 ± 3.78 and 42 ± 4.23and zeta potentials of 11 ± 2.27 and 29 ± 4.31 (mV), respectively. The polydispersity index (PI) is a parameter used to investigate the homogeneity in the particle size distribution of synthesized NPs, PI values < 0.3 guarantees the stability of colloidal dispersion.^[39]^ The size distributions of the CMD-TCs-NPs and CMD-TCs-NPs were 0.27 ± 0.05 and 0.21 ± 0.05, respectively. As demonstrated by the SEM images [Figures 3A & 3B], CMD-TMC-NPs and CMD-TCs-NPs were spherical in shape.

### Uptake of Cy5-Labeled Nanoparticles By Y79 Cells

**Figure 4:**
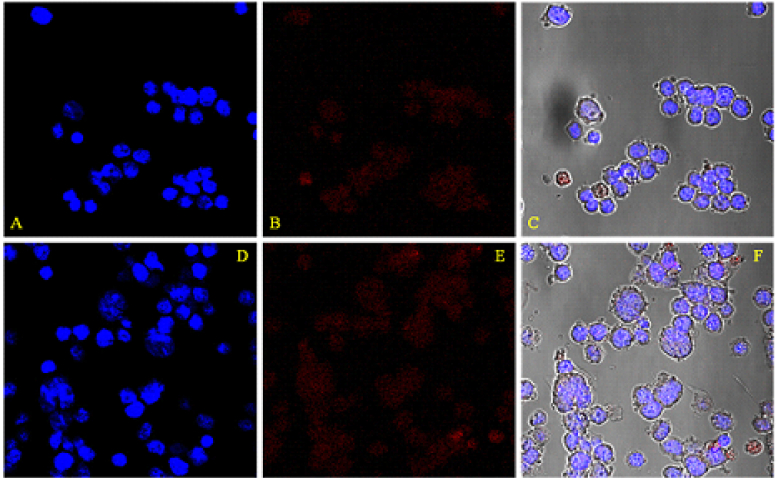
Intracellular localization of CMD-TMC-NPs (A–C) and CMD-TCs-NPs (D–F) in Y79 cells by Cy5-labeled NPs. Labeled NPs appear in red in the confocal microscopy fluorescence images.

The cellular uptake of Cy5-labeled CMD-TCs-NPs and CMD-TMC-NPs by Y79 cells was visualized using a confocal microscope after 2 hr of exposure [Figure 4]. Meanwhile, the nuclei of the Y79 cells were stained by DAPI (blue fluorescence) in order to ascertain the location of internalized NPs. A direct indicator of uptake enhancement by the Y79 cells could be increasing the number of uptakes of NPs, which was documented by the increase in the intensity of the red color as seen in Figure 4. In the Cy5-labeled NPs groups, the red signal that appeared, was mostly located in cytoplasm. Contrastingly, a stronger red signal was discovered to be distributed inside the cells treated with CMD-TCs-NPs. Compared with CMD-TMC-NPs, more bioadhesive CMD-TCs-NPs were better adsorbed by cell membrane, resulting in improved endocytosis of Y79 cells and efficacious cellular uptake [Figure 4].

### Uptake with Flow Cytometry

**Figure 5:**
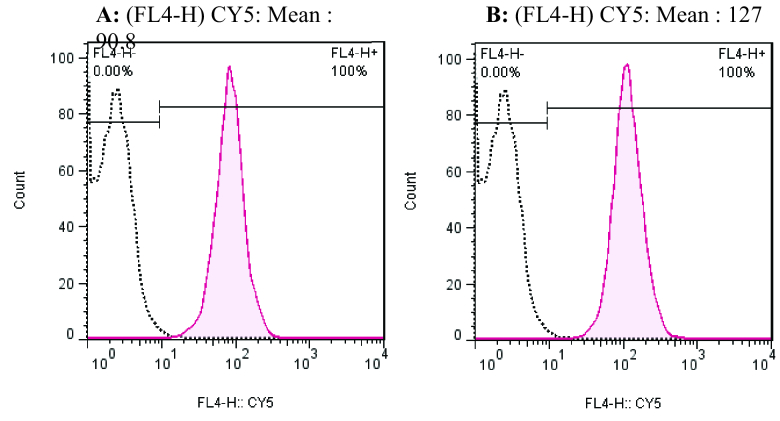
Flow cytometry analysis of cellular uptake of Cy5-labeled (A) CMD-TMC-NPs and (B) CMD-TCs-NPs in Y79 cells after 2 hr incubation time.

A rapid method for the determination of the absorption of nanoparticles in Y79 cells using flow cytometry has been used in this research. The cellular uptake of Cy5-labeled CMD-TCs-NPs and CMD-TMC-NPs by Y79 cells was further investigated by flow cytometry analysis. The fluorescence intensity of cell emission determined by flow cytometry can be a good marker of the amount of NPs internalized by Y79 cells. As shown in Figure 5, the peak of the fluorescence intensity shifted to a higher level when the CMD-TCs-NPs were used, suggesting the promoted Cy5-labeled CMD-TCs-NPs internalization by Y79.

**Figure 6:**
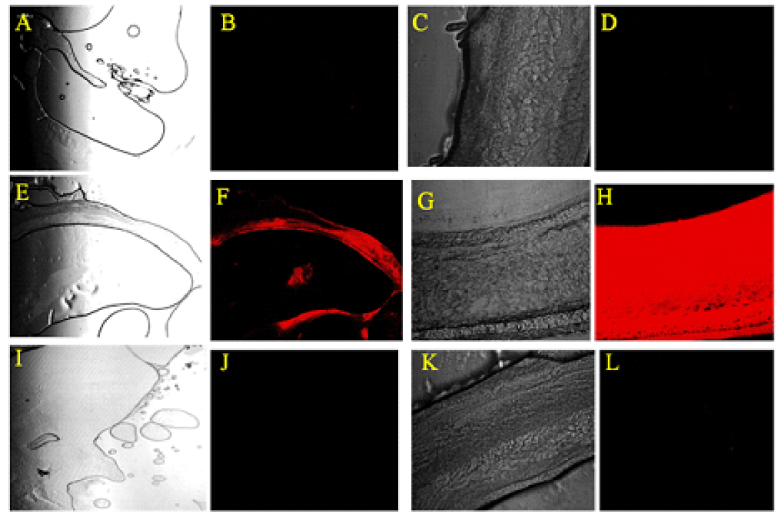
Confocal microscopy of the retinoblastoma and retina at 24 hr after intravitreal injection of CY5-labeled NPs into the vitreous cavity of rat with retinoblastoma. (A–E) Control group; untreated retinoblastoma (A) and untreated retina (C). (E–H) Eyes injected with Cy5-labeled CMD-TCs-NPs. The Cy5-labeled CMD-TCs-NPs diffused to the tumor mass (F) and through the retinal layers (H). (I–L) Eyes injected with Cy5-labeled CMD-TMC-NPs. Cy5-labeled CMD-TMC-NPs are entirely trapped in the vitreous and are not able to reach the retina even after 24 hr.

### Animal Model Diffusion Study

Within the first 24 hr after the IVT injection, the eyes were enucleated and severed into 5 μm thick sections. The NPs distribution was investigated by taking confocal images after the IVT injection of the Cy5-labeled NPs [Figure 6]. The two chitosan compositions showed various diffusion rates in the vitreous. After the injection, only Cy5-labeled CMD-TCs-NPs freely disseminated all over the vitreous cavity; 24 hr after the IVT injection, confocal microscopy demonstrated that the Cy5-labeled CMD-TCs-NPs had accumulated throughout the different retinal layers [Figure 6]. Also, it showed that cationic CMD-TCs-NPs with Zeta potentials +11 ± 2.27 mV were able to penetrate efficiently into the rat retina, while CMD-TMC-NPs with zeta potential value of +29 mV were trapped in the vitreous. The distinguished diffusion rate between the groups receiving CMD-TMC-NPs versus CMD-TCs-NPs might be due to the difference in surface charges of NPs.

##  DISCUSSION

Chemotherapy by nanoparticles has been an effective approach in ophthalmic research in overcoming poor intraocular bioavailability of drugs due to the presence of anatomical barriers and it has also contributed toward improving therapeutic efficiency. The main purpose in the engineering of nano-carriers in this investigation was to develop a promising vehicle via biopolymers to transport drugs to the posterior part of the eye. Chitosan is a polymer that has been discovered by researchers for the application of ophthalmic drug delivery systems. Mucoadhesive chitosan formulations were also considered as an effective strategy in overcoming the rapid elimination of topical ophthalmic drugs.^[40,41]^

Due to its solubility in acidic solutions (pH = 6), the efficacy of chitosan can be reduced at the site of action. Hence, a chemical alteration of chitosan was employed to fabricate a water-soluble derivative of Cs. In this study, the NPs were fabricated using hydrophilic biopolymers such as TMC, TMC-cys (TCs) and CMD to design efficient and safe drug delivery systems for the posterior segment of the eye.^[42]^ The solubility of TMC-NPs may also be decreased as a consequence of a high degree of methylation (DQ%), which results in a high level of O-methylation. The beneficial approach of combining TMC and cys to fabricate TMC-cys conjugate in preparing desirable derivatives was used in this study to improve the solubility of fabricated NPs and minimize the formation of agglomerates.^[43,44]^ Conjugation of polymers with the thiol group is the most common method used in the manufacture of mucoadhesive delivery systems.^[45]^ Endocytosis is the dominant mechanism in the adsorption of nanoparticles with a size of < 100 nm. The rate of spherical NP internalization is affected by size, shape, surface charge, composition, and surface hydrophilicity. Non-phagocytic cells absorb the highest number of spherical nanoparticles with sizes between 20 and 50 nm.^[46]^ By labeling NPs with Cy5, a qualitative assessment of their number can be obtained by evaluating the intensity of the staining seen through the confocal microscope to compare the cell uptake of the NPs. An increase in the red color intensity in the staining in the NPs-treated group, as compared to the control, could be due to better cellular uptake of NPs by Y79 cells. Therefore, the intensity of the red color that occurs after the labelling can be considered as a direct criterion for assessing the cellular uptake of NPs. As shown in Figure 4, to prove the presence of NPs in the cytoplasm, the cell nucleus was stained with DAPI. Flow cytometry was also utilized in order to provide a qualitative evaluation of the difference in cellular uptake between the two formulations of NPs. As can be seen in Figure 5, the cellular uptake of thiolated chitosan NPs by Y79 cells was better, which could be due to greater bioadhesion of TCs (TMC-cys) nanoparticles owing to the TMC combination with the thiol group.

In this research, TCs adhesion properties were founded by electrostatic interactions with cysteine. When the NPs are intravitreally injected, they must be able to cross the vitreous barrier to reach their destination. The vitreous body is a polyanionic gel-like mass which is made up of collagen fibers and glycosaminoglycan.^[47]^ Pitkänen et al showed that the major obstacle to nonviral gene delivery systems is the vitreous.^[48]^ Peeters et al.^[49]^ also stated that only PEGylated particles < 500 nm are able to have unrestricted movement through the vitreous. Later, it was declared that cationic liposomes with zeta potentials below +20 mV were allowed to defuse efficiently into the murine retina, while liposomes with zeta potential value above +20 mV were completely trapped in the vitreous humor.^[50,51]^

The drug bioavailability is dependent on the route of drug administration into the eye. Effectiveness of systematically administrated drug vehicles for ocular posterior segment drug delivery is limited by different factors including the wide drug distribution to the off-target sites and the presence of the blood–retinal barrier.^[52]^ Therefore, in this study, in order to achieve maximum bioavailability, IVT injection of nanoparticles has been used. CMD-TMC and CMD-TCs nanoparticles with identical sizes and diverse surface charges were employed to examine the connection between their diffusion rates and their composition. Confocal imaging was used to track real-time diffusion in the vitreous cavity of the injected Cy5-labelled nanoparticles. By comparing the images of the CMD-TMC-NPs, CMD-TCS-NPs, and the control groups, the Cy5 signal of labeled NPs was determined, and the variations of fluorescence intensity in the confocal images illustrated the distribution of Cy5-labelled NPs [Figure 6]. Two biocopolymers (TMC and TCs) could self-assemble into NPs with identical sizes and various surface charges. TCs could self-assemble into 34 nm NPs and with +11mV surface charges, while TMC NPs assembled into 42 nm NPs with a +29 mV zeta potential. The difference in the surface charges between TMC and TCs NPs may result in different diffusion rates of NPs after the IVT injection. In the two polymer confocal images of Figure 6, TCs with the 11 ± 2.27 mV surface charges displayed the most noticeable alterations in fluorescence signals (red color). The results of this study indicated that the surface charge of nanoparticles might perform a negative or positive role in influencing the retinal penetration of intravitreally injected NPs. This investigation also proved that vitreous with anionic properties is a weak barrier for the movement of NPs with zeta potentials +11 ± 2.27mV, but vitreous can remarkably limit TMC- diffusion with zeta potentials of +29 ± 4.31mV in vivo [Figure 6]. TMC-NPs with zeta potentials of +29 ± 4.31 mV are completely immobilized in the vitreous via electrostatic interactions with negatively charged hyaluronic acid and collagen fibers. As shown in Figure 6, methylated chitosan nanoparticles are completely trapped in the vitreous and are unable to reach the retina even after 24 hr. Thus, a powerfully positive charge (+29 ± 4.31 mV) on the particle surface has a remarkable negative impact on diffusion after IVT injection.

Through comparing the fluorescence changes in the eye injected with TCs or TMC, the results illustrated in Figure 6 indicate that the appropriate surface charges on the particle surface (i.e., +11 ± 2.27 mV) improved the diffusion efficacy of the particles after IVT injection. Accordingly, surface improving of the NPs with the thiol group in CMD-TCs-NPs boosts the transfection efficacy of the NPs through the development of intra-chain disulphide bonds within the complex. After the success of this elementary step, we plan to use nanoparticle-based CMD-TCs as a controlled drug delivery system for anticancer drugs for retinoblastoma treatment.

In conclusion, an effort to facilitate the application of more potent medicine for the local chemotherapy of retinoblastoma to ensure minimization of local and systemic adverse effects, we embarked on the current study. One challenge that is evident in delivering the relevant medicine to the posterior of the eye is the ability of the medicine to break through the vitreous cavity. Nanoparticles have been determined to be a viable alternative in targeting specific areas for medication due to their ability to adsorb and diffuse medication in specific areas locally. Our study has revealed that appropriate surface charges (preferably +11 ± 2.27 mV) on the surface of nanoparticles NPs that were manufactured with different chitosan derivatives may boost their diffusion after IVT injection. A positive charge adversely affects the rate of vitreous diffusion of NPs. This research showed that when NPs were intravitreally injected, the surface, charge of NPs is the most significant limiting factor in their penetration through the vitreous. The cationic bio-polymer with appropriate surface charges is able to reach the retina and diffuse through the retinal layers. The ionic gelation technique was efficient in synthesizing a biocompatible polymeric nanosystem for drug delivery into the posterior segment of the eye. The current study demonstrated increased ocular bioavailability of CMD-TCs-NPs relative to CMD-TMC-NPs in retinoblastoma containing rat eyes.

##  Financial Support and Sponsorship

This research was funded by Tehran University of Medical Sciences, Tehran, Iran.

##  Conflicts of Interest

The authors report no conflicts of interest in this work.
