# Erratum: Biodistribution of Cy5-labeled Thiolated and Methylated Chitosan-Carboxymethyl Dextran Nanoparticles in an Animal Model of Retinoblastoma

**DOI:** 10.18502/jovr.v17i2.10826

**Published:** 2022-04-29

**Authors:** Elham Delrish, Fariba Ghassemi, Mahmoud Jabbarvand, Alireza Lashay, Fatemeh Atyabi, Masoud Soleimani, Rassoul Dinarvand

In the article titled “Biodistribution of Cy5-labeled Thiolated and Methylated Chitosan-Carboxymethyl Dextran Nanoparticles in an Animal Model of Retinoblastoma" published on pages 58–68, Volume 17, Issue 1 of *Journal of Ophthalmic and Vision Research*,^[1]^ the statement for Ethical Consideration was missing in the full-text PDF and HTML of the article. The online version of the article has therefore been updated on April 29, 2022 and can be accessed from https://doi.org/10.18502/jovr.v17i1.10171.

##  Ethical Consideration

The study protocol was approved by Farabi Eye Hospital, Tehran University of Medical Sciences Ethics Committee. The ethical code is “IR.TUMS.FARABIH.REC.1398.002”. The study has followed National Ethical Guidelines. All surveys conformed to the Association for Research in Vision and Ophthalmology (ARVO) statement for the use of animals in research.
